# The Impact of Metabolic Syndrome and Lifestyle Habits on the Risk of the First Event of Cardiovascular Disease: Results from a Cohort Study in Lithuanian Urban Population

**DOI:** 10.3390/medicina56010018

**Published:** 2020-01-03

**Authors:** Vilma Jasiukaitienė, Dalia Lukšienė, Abdonas Tamošiūnas, Ričardas Radišauskas, Martin Bobak

**Affiliations:** 1 Laboratory of Population Studies of Institute of Cardiology, Medical Academy, Lithuanian University of Health Sciences, LT-50162 Kaunas, Lithuania; dalia.luksiene@lsmuni.lt (D.L.); abdonas.tamosiunas@lsmuni.lt (A.T.); ricardas.radisauskas@lsmuni.lt (R.R.); 2 Department of Environmental and Occupational Medicine, Faculty of Public Health, Medical Academy, Lithuanian University of Health Sciences, LT-47181 Kaunas, Lithuania; 3 Department of Preventive Medicine, Faculty of Public Health, Medical Academy, Lithuanian University of Health Sciences, LT-47181 Kaunas, Lithuania; 4 Department of Epidemiology and Public Health, University College London, London WC1E 6BT, UK; m.bobak@ucl.ac.uk

**Keywords:** metabolic syndrome, nutrition habits, smoking, physical activity, cardiovascular diseases

## Abstract

*Background and Objectives:* In recent years, the impact of individual risk factors on mortality from cardiovascular diseases (CVD) has been often investigated. However, there is a lack of studies that have evaluated the relationship between lifestyle habits, metabolic syndrome, and their combined influence on the first event of CVD. The aim of this study was to investigate the impact of metabolic syndrome and lifestyle habits on the risk of the first event of CVD in a Lithuanian urban population. *Materials and Methods:* The presented data were collected from a survey that was carried out within the framework of the international project Health, Alcohol and Psychosocial Factors in Eastern Europe (HAPIEE). For statistical analysis, 4257 participants aged 45–72 years were selected (with a follow-up of 11 years). *Results:* The findings from the Cox proportional hazards regression multivariable analysis showed that metabolic syndrome, current smoking status, and former smoking status increased the risk of the first event of CVD among men (with respective hazard ratios (HR) of 1.53, 1.94, and 1.43; *p* < 0.01). In women, metabolic syndrome increased the risk of the first event of CVD (HR = 1.56; *p* = 0.001), while the increased consumption of fresh vegetables and fruits decreased the risk of the first event of CVD (HR = 0.80; *p* = 0.003). Multivariable logistic regression analysis results show that a level of increased physical activity by one hour can be linked to a lower risk of metabolic syndrome by 2% among men (odds ratio (OR) = 0.98; *p* = 0.001). *Conclusions:* Metabolic syndrome and lifestyle habits including cigarette smoking in men and low consumption of fresh vegetables and fruits in women are strong predictors of the first event of CVD.

## 1. Introduction

The 2018 aging report that analyzed the European policy challenges for aging societies showed that fiscal costs linked to pensions, health care and long-term care are expected to rise over the coming decades as Europe’s population continues to age significantly [1]. Thus, if we want to achieve healthy aging, it is necessary to identify and understand the determinants of human health and disease, as well as risk factors for diseases over time [2]. As reported by the World Health Organization (WHO), cardiovascular diseases (CVD) have become one of the biggest threats to people’s health [3]. For instance, in Lithuania, the incidence and mortality rates of CVD are higher than in most European countries [4,5]. For this reason, it is very important to determine the complex impact of major risk factors that may be associated with the incidence of the first event of CVD. Our previous studies analyzed the impact that separate factors, such as metabolic syndrome and lifestyle habits, such as physical activity, have on mortality from CVD. As a result, it was discovered that metabolic syndrome increased the risk of mortality from CVD among Lithuanian men [6], while physical activity in leisure time had a preventive effect [7]. Nevertheless, the data from a long-term cohort study conducted in Lithuania showed that smoking clearly increased the risk of all-cause mortality in males aged 35–64 years, as well as CVD mortality [8]. As can be seen, many studies in recent years have analyzed the effects of individual lifestyle risk behaviors on mortality from CVD. However, there has been a lack of studies carried out, especially in Eastern European countries, that have evaluated the relationship between lifestyle habits, metabolic syndrome, and their impact on the lifetime incidence of the first event of CVD in the urban adult and elderly population. We hypothesized that metabolic syndrome and lifestyle habits are strongly and directly associated with the risk of the first event of CVD and have an additive interaction. This study aimed to investigate the impact of metabolic syndrome and lifestyle habits (such as smoking status, nutrition habits and physical activity level) on the risk of the first event of CVD in the Lithuanian middle-aged and elderly urban population.

## 2. Materials and Methods

### 2.1. Study Sample

The presented data were collected from a survey that was carried out within the framework of the international project Health, Alcohol and Psychosocial Factors in Eastern Europe (HAPIEE) [9]. The baseline survey was conducted during 2006–2008. The 10,980 individuals were randomly selected for the study from the National Population Register. A group of 7115 urban men and women from Kaunas city (Lithuania) aged 45–72 years participated in this survey. In the study, the response rate was 64.8%. A study sample was stratified by sex and age. 4257 participants in total (2076 men and 2181 women) were available for statistical analysis after applying the exclusion criteria. First, we excluded those responders who previously had CVD (*n* = 1556). Second, we excluded those respondents who received pharmacological treatment for diabetes and/or dyslipidemia, as well as those who were treated with a diet as recommended by a doctor (*n* = 1032). Third, some participants were excluded because the metabolic syndrome component information was incompletely provided (*n* = 270). 

The study protocol was approved by the Ethics Committee at the University College London, UK and by the Kaunas Regional Biomedical Research Ethics Committee, Lithuanian (11 January 2005; No. 05/09). All participants signed an informed consent form.

### 2.2. Sociodemographic and Lifestyle Factors

During the study, sociodemographic, lifestyle factors and nutritional habits data were collected with a standardized questionnaire. The questionnaire included questions regarding the respondent’s sociodemographic factors, such as sex, age, and educational status. 

The questionnaire also evaluated lifestyle factors, such as smoking status, nutrition habits and physical activity. Smoking habits were categorized by classifying the participants into three groups: current smokers, former smokers, and never-smokers. Moreover, physical activity was determined by the mean length of time spent per week during leisure time in winter and summer for walking, moderate and hard work like gardening and other physical activities. The respondents were categorized into two groups according to their leisure time physical activity: physically active (10 h or more) and inactive (<10 h). 

Nutrition habits were evaluated by using a food frequency questionnaire. The questionnaire was adapted and validated for the Lithuanian population. Twenty food groups were included in the food frequency questionnaire: potatoes, porridges and cereals, cheese, curd cheese, chicken, fish, meat, sausage, eggs, fresh carrots (in summer and autumn and in winter and spring), other fresh vegetables (in summer and autumn and in winter and spring), boiled vegetables, fresh fruit (in summer and autumn and in winter and spring), natural juice, candies, chocolate, and cakes. In the questionnaire, there were six possible responses for each food group: every day; 4–6 times per week; 2–3 times per week; once per week; 2–3 times per month; and rarely or never. Higher values denoted a more frequent use of the current food. An exploratory factor analysis was used to reduce the number of food items (see Section 2.4.).

### 2.3. Biochemical Indicators and Diagnostic Criteria of the Metabolic Syndrome

For the participants, blood pressure was measured three times with an oscillometric device (Omron M5-I), and the collected average values were used for the analysis. Waist circumference was measured (without upper clothes) by a standard type meter with an accuracy of 0.5 cm. 

Fasting blood serum samples were analyzed in the WHO Regional Lipid Reference Centre, Institute of Clinical and Experimental Medicine, Prague (Czech Republic). Lipid concentrations (triglycerides and high-density lipoprotein (HDL) cholesterol) in serum were measured on a Roche COBAS MIRA auto-analyzer with the use of a conventional enzymatic method with reagents from Boehringer-Mannheim Diagnostics and Hoffmann-La Roche. The WHO Regional Lipid Reference Centre was responsible for the quality control of biochemistry measures. The concentration of glucose in capillary blood was determined by an individual Glucotrend glucometer [10]. 

The diagnostic criteria for metabolic syndrome by the Third Report of the National Cholesterol Education Program Adult Treatment Panel III (NCEP-ATP III) definition are the presence of three or more of the following risk determinants: 1) increased waist circumference (≥102 cm for men and ≥88 cm for women); 2) elevated triglycerides (≥1.7 mmol/L); 3) low HDL cholesterol (<1.0 mmol/L in men and <1.3 mmol/L in women); 4) arterial hypertension (≥130/85 mmHg); and 5) impaired fasting glucose (≥6.1 mmol/L) [11].

### 2.4. Statistical Analysis

Statistical analyses were performed with the IBM SPSS statistics 20.0 software. The analysis was performed separately for men and women. The prevalence of lifestyle habits and risk factors was compared in gender groups and in groups with/without the first event of CVD via χ^2^ tests. The mean differences in these groups were tested by the *t*-test. Proportions were compared by using *z* tests. The difference was considered to be statistically significant when *p* < 0.05.

Data on exploratory factor analysis were presented in our previous paper [12]. For the simplicity of data interpretation, only factor loadings of ≥0.4 are presented in Table 1. The consumption of meat and meat products was conversely correlated with the consumption of cereals and porridge. A dichotomous dependent variable was constructed by dividing the factor scores into two groups (1—more frequent than average consumption of a particular food group; 0—less frequent than average consumption).

Hazard ratios (HR) and 95% confidence intervals (CI) were estimated by the multivariate Cox proportional hazards regression for the first event of CVD (non-fatal or fatal ischemic heart disease (IHD) and stroke). The proportionality of hazards, including time-dependent covariates in the Cox models, was testing. All variables included in the models met the risk proportionality assumption. To collect the data, several models were assessed. Firstly, Model 1 was adjusted for age. Secondly, Model 2 was adjusted for age, metabolic syndrome, nutrition factors, smoking status, physical activity and education level. A multivariable logistic regression analysis was performed to determine the independent associations between physical activity and metabolic syndrome, and its components were expressed as an odds ratio (OR) with 95% CI. According to the authors Skosyrev and Glimm [13], we calculated the power of statistically significant variables in multivariable Cox regression Model 2.

## 3. Results

The study participants were followed-up from the beginning of the baseline health examination date until 31 December 2017. Non-fatal CVD events and mortality data were collected from the Kaunas population-based Ischemic Heart Disease (IHD) and Stroke Registers and the official mortality registry. Additionally, the outcomes were measured as the first non-fatal CVD event and cases of death from CVD (excluding those with a documented history of CVD at entry). The mean duration and SD of follow-up were 8.46 ± 2.51 years among men and 9.06 ± 1.85 years among women. Mortality from CVD events included death from IHD, stroke, and other vascular causes; these were defined as I00–I99 codes by the 10th International Classification of Diseases (ICD). Non-fatal CVD cases included the first events of IHD (definite and possible acute myocardial infarction according to the criteria of the Multinational Monitoring of Trends and Determinants in Cardiovascular Disease (MONICA) project) and stroke (according to the criteria of MONICA project) [14]. There were 118 deaths from CVD (excluding those with the previous CVD at entry) (83 men and 35 women). During the follow-up period, there were 227 identified (152 men and 75 women) cases of first non-fatal events of IHD and 113 (63 men and 50 women) events of stroke. The total number of first CVD cases was 458 (10.8%).

The characteristics of the respondents at the baseline survey according to the first event of CVD are presented in Table 2. As a result, men and women who had their first event of CVD during the follow-up period were older, less educated, and had a higher incidence rate of metabolic syndrome. In addition, it was determined that the respondents who had their first event of CVD had a higher arterial hypertension rate, higher elevated triglycerides, lower HDL cholesterol rates, and more increased waist circumference rate than those who did not have any CVD events. Meanwhile, men who had their first event of CVD had a higher proportion of smokers than those without CVD events. Statistically significant differences were registered in the nutrition habits between the respondents who had their first event of CVD compared to those without CVD events. For example, women ate fresh vegetables and fruits less frequently; men consumed sweeteners less frequently (*p* < 0.01). 

We evaluated the risk of the first event of CVD concerning the presence of metabolic syndrome, lifestyle habits, and education (Table 3). To begin with, the first event of CVD concerning each of these variables and age was analyzed (Model 1). The results showed that metabolic syndrome increased the risk of the first event of CVD by 57% on average among men and women (*p* < 0.01). Additionally, current and former smoking status significantly increased the risk of the first event of CVD among men (on average by 104% and 54%, respectively) (*p* < 0.01). In comparison, high physical activity decreased the risk of the first event of CVD by 22% on average among men (*p* = 0.043). Moreover, it was determined that increased consumption of fresh vegetables and fruits (first nutrition factor “fresh vegetables and fruits”) decreases the risk of the first event of CVD by 19%, on average, only among women (*p* = 0.003). 

Next, by using the multivariable Cox model, we evaluated the effect of age, education level, metabolic syndrome and lifestyle habits (i.e., nutrition factors, smoking, and physical activity level) on the first event of CVD (Model 2). The results showed that only metabolic syndrome, current smoking status, and former smoking status significantly increased the risk of the first event of CVD among men (on average by 53%, 94%, and 43%, respectively). As for the women, the metabolic syndrome increased the risk of the first event of CVD by 56% on average (*p* = 0.001), whereas the increased consumption of fresh vegetables and fruits (first nutrition factor “fresh vegetables and fruits”) decreased the risk of the first event of CVD by 20% on average (*p* = 0.003). However, it was identified that physical activity and education levels were not directly related to the first event of CVD in men and women. We calculated the power of statistically significant variables in multivariable Cox regression Model 2 (Table 3). In the male group, the power for metabolic syndrome was 95% (second type error = 0.05); for current smoking, it was 99% (second type error = 0.01); and for former smoking, it was 69% (second type error = 0.31). In the female group, the power for metabolic syndrome was 78% (second type error = 0.22); and for the “fresh vegetables and fruits“ factor, the power was 83% (second type error = 0.17). 

Therefore, after additional multivariable logistic regression analysis, the results showed that the increase of physical activity level by one hour could be associated with a lower risk of metabolic syndrome by 2% among men (OR = 0.98; 95% CI 0.98–0.99; *p* = 0.001). Furthermore, physically active men had lower odds of arterial hypertension (OR = 0.70; 95% CI 0.54–0.91; *p* = 0.008), of larger waist circumference (OR = 0.78; 95% CI 0.63–0.97; *p* = 0.023), and of increased glucose level (OR = 0.72; 95% CI 0.59–0.88; *p* = 0.002) in comparison with physically inactive men. Physically active women had lower odds of increased glucose levels in the blood (OR = 0.78; 95% CI 0.62–0.99; *p* = 0.046) in comparison with physically inactive women. 

## 4. Discussion

Healthy aging is largely determined by individual lifestyle choices [15]. Despite the favorable changes in some risk factors and mortality rates in the Lithuanian middle-aged population during 1985–2013, the prevalence of risk factors such as smoking, arterial hypertension, obesity, and mortality from CVD in Lithuania are still high [16]. 

In our large longitudinal study, we analyzed the impact of metabolic syndrome and lifestyle habits on the risk of the first event of CVD. In addition, the study findings reported that metabolic syndrome and some lifestyle habits, such as smoking and fresh vegetable and fruit consumption, are strongly associated with the first event of CVD. The results from Italian Rural Areas of the Seven Countries Study of Cardiovascular Diseases showed that no smoking, vigorous activity, and the Mediterranean diet were beneficial or protective for the lifetime incidence of coronary heart disease, while the reverse effect was true for heavy smoking, sedentary habits, and the non-Mediterranean diet [17]. 

However, the more detailed results of our study show that metabolic syndrome is one of the most important risk factors for the first event of CVD. Multivariable analysis methods support the idea of a strong relationship between metabolic syndrome and the risk of the first event of CVD among men and women. To be more precise, metabolic syndrome increases the risk of the first event of CVD by more than 50%. Additionally, numerous studies have shown the relationships between metabolic syndrome and an increased risk of mortality from CVD [6,18,19]. However, it can be noted that there are a lack of data that could allow for the analysis of the impact of metabolic syndrome on the first event of CVD. 

Many reports have dealt with the importance of lifestyle behaviors, such as smoking, and their impact on the risk of non-communicable diseases [20,21]. The effect of the current and former smoking status can act as very important risk factors for morbidity and mortality from CVD [21]. The results of the previously conducted study showed that smoking clearly increased the risk of all-cause mortality and mortality from CVD of the Lithuanian male population aged 35–64 years [8]. The results from this study confirmed and specified the results of the previous study, showing that current and former smoking status significantly increases the risk of the first event of CVD among men (respectively, by 94% and 43% on average). In addition to the fact that smoking increases the first event of CVD, metabolic syndrome is another strong risk predictor that was included in the same research model (Model 2). Furthermore, in the women’s group, there were no significant associations between smoking status and the first event of CVD. The main reason for this was that the number of women who were current or former smokers was limited and the prevalence of regular smoking in women was 2.8 times lower when compared to men. The same finding was disclosed in the results of another research project from the Seven Countries Study: Smoking was strongly associated with mortality and survival in middle-aged men during the 40-year follow-up [22]. 

When epidemiologists and scientists prepared the 2016 European Guidelines on cardiovascular disease prevention in clinical practice [21], they unanimously agreed that some lifestyle habits, such as healthy nutrition habits and physical activity, have a protective role in morbidity and mortality from CVD, mainly due to their positive impact on risk factors. For this reason, in this study, complex risk factors, such as metabolic syndrome, dietary habits, and physical activity, that could result in the first event of CVD were analyzed. What is more, nutrition habits have been found to be the most important lifestyle determinant for all-cause mortality risk. Several research studies have discussed the relationship between nutrition habits and the risk of morbidity and mortality from CVD and cancer [23,24,25,26]. Recently, a lot of attention has been paid to research studies on vegetables and fruits and their influence on human health [23,27]. In this study, nutrition habits were analyzed by applying an exploratory factor analysis. The results of our study show that an increased consumption of fresh vegetables and fruits (first nutrition factor “fresh vegetables and fruits”) decreases the risk of the first event of CVD by 20% on average among women. Multivariable analysis methods support the idea of a strong relationship between an increased consumption of fresh vegetables and fruits with a decreased risk of the first event of CVD in women. Metabolic syndrome was also included in the same risk prediction model (Model 2). In the men’s group, there were no significant associations found between nutrition habits and the first event of CVD, and a possible reason for this might be that smoking is the most important lifestyle behavior risk factor. Moreover, numerous observational epidemiological studies have found inverse associations between vegetable and fruit intake and the risk of CVD [23,25]. A conducted meta-analysis of 95 studies observed that vegetable and fruit intake can be associated with reduced risk of CVD, cancer, and all-cause mortality [23]. Additionally, the cardioprotective effects of vegetables and fruits might involve antioxidation; anti-inflammation that regulates blood pressure, blood glucose, and lipid profiles; attenuating myocardial damage and modulating relevant enzyme activities; and some other biomarkers associated with CVD [27]. Besides, some studies have focused on cereal fiber intake and the risk of mortality from all causes, CVD, cancer, and inflammatory diseases [26]. In our study, an exploratory factor analysis indicated the second factor as “increased consumption of meat and low of cereals.” Unfortunately, there were no significant associations found between those nutrition habits and the risk of the first event of CVD. It can be stated that one of the possible explanations for this could be that an increased consumption of meat and low of cereals is more prevalent in men in comparison with women, and the most crucial risk factor of men’s lifestyle behavior is regular smoking.

A generally active daily life is associated with cardiovascular health and longevity in older adults [28]. Previous studies have indicated that lifestyle behavior linked to physical activity is strongly associated with mortality and survival rates in the middle-aged population [17,22,28]. The results of our study showed that physical activity was not directly related to the first event of CVD among men and women. Though our analysis supports the idea of the strong relationship of physical activity with the first event of CVD in men, after multivariable analysis, an intriguing finding was the absence of a significant association between physical activity and CVD incidence. In contrast, a strong significant association was found between metabolic syndrome and physical activity. The results from our study indicate that a level of increased physical activity by one hour is associated with a lower risk of metabolic syndrome by 2% among men and by 1% among women. The results of the study conducted in Taiwan showed that physical activity is associated with a lower incidence of several common biological CVD risk factors: obesity, atherogenic dyslipidemia, metabolic syndrome, and type 2 diabetes [29]. The results of the systematic meta-analysis (16 articles, 18 studies including 76,699 participants and 13,871 cases of metabolic syndrome) provided quantitative data that suggested that any amount of physical activity during leisure time is better than none. This is associated with an additional reduction in metabolic syndrome risk [30].

According to our results, general practitioners who diagnose one or more components of metabolic syndrome should inform the patient about the increased risk of the first event of CVD. However, they also should stress that consuming more vegetables and fruits (especially for women) and the cessation of smoking (especially for men) can reduce the likelihood of these events. Those recommendations are actually viable for various age groups of the population. Therefore, efforts to support a healthy lifestyle should be a public health priority in Lithuania. 

### 4.1. Strengths

The strength of this study is that it included a large number of middle-aged and elderly participants of the Lithuanian population and a long-term follow-up. It should be noted that the data on patients with or without cardiovascular diseases were collected from Kaunas Ischemic Heart Disease and Stroke Registers for the determination of endpoints (i.e., first events). In addition, this is the first long-term epidemiological study on the combined impact of lifestyle habits and metabolic syndrome on the risk of the first event of CVD in Baltic countries. Moreover, multiple lifestyles and biological CVD risk factors were assessed with the use of uniform and standardized data collection methods. This allowed us to make adjustments for a large number of potential confounding variables in the Cox models.

### 4.2. Limitations

There are some limitations to our study. The method of the factor analysis for the identification of dietary patterns presents some limitations. The results depended on the food items included, the number of the factors extracted, and the method of rotation used. Additionally, we used a food frequency questionnaire with a semi-quantitative scale. The limitation of the categorical food-frequency scale is that the answers may have been affected by subjective evaluation of the participants and may have been less accurate than quantitative methods for calculating food servings (for example, one day of 24-h dietary recall; seven days’ diet diary). It can be suggested that these findings should be interpreted cautiously, as the factor structure depended on the food items that were initially assessed. Another limitation is that capillary blood glucose was measured; for this reason, it is likely that our measures were less reliable and less sensitive in detecting metabolic syndrome. The next problem is that the family history of CVD was not included in the multivariate Cox proportional hazards regression model.

## 5. Conclusions

Metabolic syndrome and lifestyle habits, including smoking in men and a low consumption of fresh vegetables and fruits in women, are strong predictors of the lifetime incidence of the first event of CVD. Physical activity is related to a lower metabolic syndrome risk among men. According to our results, future efforts to support a healthy lifestyle should be a public health priority in Lithuania.

## Figures and Tables

**Table 1:** Factors and factor loadings for the dietary variables.

Type of Food	1st Factor	2nd Factor	3rd Factor	4th Factor	5th Factor
	Fresh Vegetables, Fruit	Increased Consumption of Meat and Low Cereals	Sweets	Boiled Vegetables and Potatoes	Chicken, Fsh, and Eggs
Fresh vegetables	0.739				
Fresh fruit	0.697				
Fresh carrots	0.485				
Natural juice	0.439				
Cereals, porridge		−0.651 *			
Meat products (sausage)		0.651			
Meat		0.638			
Sweets			0.848		
Sweet pastries			0.845		
Potatoes				0.693	
Boiled vegetables				0.626	
Chicken					0.711
Fish					0.594
Eggs					0.447

Factor loadings of <0.4 were excluded from the table for simplicity. * Negative values of factor loadings indicate a low consumption of cereals.

**Table 2:** Baseline characteristics of the study population according to the first event of cardiovascular diseases (CVD).

Variables	Men		Women	
	The First Event of CVD		The Frst Event of CVD	
	No	Yes	No	Yes
	*N* = 1778	*N* = 298	*N* = 2021	*N* = 160
Mean age, years (SD)	59.2 (7.6)	62.5 (7.2) ***	58.6 (7.7)	63.6 (6.8) ***
Metabolic syndrome (%)	20.9	29.5 ***	29.4	44.4 ***
Mean metabolic syndrome components (SD)	1.7 (1.1)	2.0 (1.2) ***	1.9 (1.4)	2.4 (1.3) ***
Increased waist circumference (%) (for men ≥102 cm; for women ≥88 cm)	23.1	35.2 ***	46.4	60.6 ***
Elevated triglycerides level (%) (≥1.7 mmol/L)	23.1	29.9 **	22.4	30.6 *
Low HDL cholesterol level (%) (for men <1.0 mmol/L, for women <1.3 mmol/L)	9.4	13.1 *	19.9	35.0 ***
Increased fasting glucose level (%) (≥6.1 mmol/L)	27.4	28.9	28.4	25.6
Arterial hypertension (%) (>130/85 mm Hg)	82.3	89.6 ***	68.7	83.8 ***
Smoking status (%)				
Never	41.3	32.2 **	81.4	85.6
Former	27.1	31.2	7.0	3.8
Current	31.6	36.6	11.6	10.6
Physically active (%)	69.1	67.1	82.5	83.1
Education level (%)				
Primary	5.4	10.7 **	5.2	13.8 **
Vocational	8.6	15.8 ***	7.7	10.0
Secondary	32.3	28.5	24.5	22.5
College	18.8	15.8	27.6	30.6
University	34.9	29.2	35.0	23.1 **
Nutrition factors (%)				
1st “Fresh vegetables and fruits”	49.5	48.0	60.2	49.4 **
2nd “Increased consumption of meat and low cereals”	66.1	61.7	37.0	31.9
3rd “Sweets”	51.0	42.6 **	53.6	56.3
4th “Boiled vegetables and potatoes”	55.5	54.7	45.7	45.0
5th “Chicken, fish, and eggs”	58.2	60.4	46.7	48.1

Mean (SD) follow-up for men was 8.46 (2.51) years; for women, it was 9.06 (1.85) years. * *p* < 0.05; ** *p* < 0.01; *** *p* < 0.001, compared to those who did not have CVD event.

**Table 3:** Risk of the first event of CVD during the 11-year follow-up by lifestyle habits in the Lithuanian urban population.

Variables	Men		Women	
	Model 1	Model 2	Model 1	Model 2
	HR (95% CI)	HR (95% CI)	HR (95% CI)	HR (95% CI)
Metabolic syndrome	1.57 (1.23–2.02)	1.53 (1.18–1.97)	1.57 (1.15–2.15)	1.56 (1.14–2.15)
Nutrition factors				
1st “Fresh vegetables and fruits”	0.91 (0.81–1.01)	0.95 (0.85–1.07)	0.81 (0.70–0.93)	0.80 (0.69–0.93)
2nd “Increased consumption of meat and low cereals”	1.06 (0.93–1.20)	0.98 (0.86–1.12)	1.12 (0.95–1.31)	1.08 (0.92–1.28)
3rd “Sweets”	0.89 (0.79–1.00)	0.92 (0.82–1.03)	0.89 (0.75–1.04)	0.91 (0.78–1.07)
4th “Boiled vegetables and potatoes”	0.98 (0.87–1.10)	0.97 (0.86–1.10)	1.00 (0.86–1.16)	1.00 (0.86–1.16)
5th “Chicken, fish, and eggs”	1.05 (0.94–1.17)	1.05 (0.94–1.18)	0.99 (0.84–1.15)	0.96 (0.82–1.13)
Smoking status (%)				
Never	1	1	1	1
Former	1.54 (1.16–2.05)	1.43 (1.07–1.90)	0.90 (0.39–2.06)	0.92 (0.40–2.11)
Current	2.04 (1.54–2.72)	1.94 (1.45–2.60)	1.67 (0.98–2.85)	1.58 (0.92–2.73)
Physical activity (%)				
Physically inactive	1	1	1	1
Physically active	0.78 (0.61–0.99)	0.85 (0.66–1.09)	0.99 (0.66–1.50)	1.13 (0.74–1.73)
Education level (%)				
Primary	1	1	1	1
Vocational	1.23 (0.78–1.94)	1.16 (0.74–1.83)	0.74 (0.39–1.41)	0.79 (0.41–1.52)
Secondary	0.78 (0.51–1.20)	0.78 (0.51–1.20)	0.82 (0.47–1.43)	0.85 (0.48–1.49)
College	0.70 (0.44–1.12)	0.76 (0.48–1.22)	1.01 (0.59–1.73)	1.21 (0.70–2.09)
University	0.66 (0.43–1.00)	0.72 (0.48–1.10)	0.61 (0.35–1.06)	0.75 (0.43–1.34)

Mean (SD) follow-up for men was 8.46 (2.51) years; for women, it was 9.06 (1.85) years. Model 1—adjusted for age. Model 2—adjusted for age, metabolic syndrome, nutrition factors, smoking status, physical activity, and education level.
